# Phacoemulsification Surgery in Eyes with Neovascular Age-Related Macular Degeneration

**DOI:** 10.1155/2014/417603

**Published:** 2014-01-22

**Authors:** Andre Grixti, Evangelia Papavasileiou, Dominic Cortis, Balakrishna Vineeth Kumar, Som Prasad

**Affiliations:** ^1^Department of Ophthalmology, Wirral University Teaching Hospital, Arrowe Park Road, Wirral, Merseyside CH49 5PE, UK; ^2^Department of Ophthalmology, St. Paul's Eye Unit, Royal Liverpool University Hospital, Prescot Street, Liverpool, Merseyside L7 8XP, UK; ^3^Department of Mathematics, University of Leicester, University Road, Leicester LE1 7RH, UK; ^4^The Insurance Unit, Faculty of Economics, Management and Accountancy, University of Malta, Msida, MSD2080, Malta

## Abstract

*Purpose*. To evaluate the visual outcomes and effect of phacoemulsification surgery on the progression of neovascular age-related macular degeneration (AMD). *Methods*. Retrospective, noncomparative, and interventional case series. Thirty eyes from 29 subjects with neovascular AMD treated with intravitreal antivascular endothelial growth factor (VEGF) injections who underwent phacoemulsification and had a postsurgery follow-up of 6 months were included. LogMAR best corrected visual acuity (BCVA) was assessed preoperatively; 1 month, 3 months, and 6 months postoperatively; and finally at the last visit. The frequency of anti-VEGF therapy, calculated as the number of intravitreal injections per month, and central macular thickness (CMT) before and after cataract surgery were determined. *Results*. Median (range) logMAR BCVA was 0.69 (0.16 to 1.32) preoperatively; 0.55 (−0.04 to 1.32) at 1 month, 0.52 (−0.1 to 1.32) at 3 months, and 0.50 (0.0 to 1.32) at 6 months postoperatively; and 0.6 (0.0 to 1.4) at final visit (*P* = 0.0011). There was no difference in the frequency of anti-VEGF injections between the immediate 6 months before and after phacoemulsification, which was equal to 0.1667 injections per month (*P* = 0.6377). Median CMT measured 203 **μ**m preoperatively, which temporarily increased to 238 **μ**m at 1 month after surgery (*P* = 0.0093) and then spontaneously returned to baseline, measuring 212.5 **μ**m at 3 months postoperatively (*P* = 0.3811). *Conclusion*. Phacoemulsification surgery significantly improved vision in patients with neovascular AMD, with no increased need for anti-VEGF injections to keep the macula dry postoperatively.

## 1. Introduction

Age-related macular degeneration (AMD) and cataract are common ocular disorders in the elderly which often develop concurrently and their prevalence is likely to increase with increasing longevity worldwide. Visually significant cataract may further compromise the vision in patients with AMD. In addition, the formation of cataract may have an impact on the possibility to evaluate the effect of treatment and performing investigations such as optical coherence tomography (OCT) or fundus fluorescein angiography. This impairs monitoring of disease progression. The possibility of a relationship between cataract surgery and progression of AMD has generated considerable debate among ophthalmologists. Several reports have been published which either support [1–8] or reject [9–17] a link between cataract surgery and deterioration of AMD. The strongest evidence supporting development of advanced AMD in the postoperative period was provided by large population-based epidemiologic studies [4–7]. However, a cochrane review of the literature was unable to determine whether cataract surgery was beneficial or harmful in patients with AMD [18]. More recently, data from the Age-Related Eye Disease Study (AREDS) concluded that cataract surgery is safe in the setting of dry AMD and did not accelerate progression to advanced sight threatening forms of AMD [16, 17].

The majority of the studies in the literature focus on the association between cataract surgery and progression of early AMD to exudative AMD. However, the impact of phacoemulsification surgery on eyes with preexisting neovascular AMD treated with intravitreal antivascular endothelial growth factor (VEGF) injections is not well known and cataract surgery may be considered controversial. Concerns include exacerbation of leakage and submacular haemorrhage resulting in further deterioration of vision, guarded visual prognosis due to macular scarring, and increased risk of perioperative complications associated with multiple previous intraocular injections [19]. Recently, a number of retrospective studies have shown a beneficial effect of cataract surgery in eyes with exudative AMD [19–21].

In the current study, we evaluate the visual outcomes, adverse events, and the effect of cataract extraction by phacoemulsification surgery on the progression of neovascular AMD in eyes treated with intravitreal ranibizumab (Lucentis) injections.

## 2. Materials and Methods

A retrospective case series was performed including all subjects with neovascular AMD treated with intravitreal anti-VEGF injections who had cataract extraction by phacoemulsification surgery between June 2007 and February 2012. The study was approved by the institutional review board and complied with the tenets of the Declaration of Helsinki. All cases were identified through a search of the electronic medical record (EMR) database at the Wirral University Teaching Hospital (Medisoft). First, the database was searched automatically for relevant diagnoses. A total of 197 subjects who received intravitreal anti-VEGF injections and had phacoemulsification surgery were detected. All eyes that had undergone vitrectomy, epimacular brachytherapy, tissue plasminogen activator (TPA)/gas injection for macular haemorrhage, or dry AMD prior to cataract surgery were excluded. Additional exclusion criteria included diabetic retinopathy, retinal vascular occlusions, high myopia, and advanced glaucoma. The individual medical records of 29 selected subjects with a preexisting diagnosis of neovascular AMD who underwent phacoemulsification surgery after initiation of anti-VEGF therapy with ranibizumab (Lucentis) and had a postsurgery follow-up of 6 months were then reviewed retrospectively. It is our practice not to discharge patients with neovascular AMD, so any patients who may have had poor visual outcomes were not excluded.

All patients had a baseline fundus fluorescein angiography confirming the diagnosis of exudative AMD. Our treatment and retreatment protocol for neovascular AMD was based on the prospective optical coherence tomography imaging of patients with neovascular AMD treated with intraocular ranibizumab (Lucentis) [PrONTO] study criteria [22]. Treatment comprised an initial loading dose of 3 consecutive monthly injections followed by optical coherence tomography (OCT) guided retreatment at monthly follow-up visits. The presence of any macular fluid or an increase in central macular thickness (CMT) of at least 100 *μ*m as detected by OCT was an indication for retreatment. Patients were listed for cataract surgery when the choroidal neovascular complex was considered stable; that is, the patient achieved an injection-free phase. This was defined as two consecutive visits (approximately 3 months) without signs of activity on OCT. The cataract was considered to be visually significant when it was at least Grade 3 on LOCS III (Lens Opacities Classification System). Phacoemulsification surgery was performed using Bausch and Lomb Stellaris vision enhancement system with an aspheric acrylic lens (Akreos MI60 lens) inserted through a 1.8 mm clear corneal microincision.

Data was recorded as part of our standard treatment protocols for wet AMD patients. The primary outcome measure was the best corrected visual acuity (BCVA) measured in Early Treatment Diabetic Retinopathy Study (ETDRS) charts and recorded in logMAR units. BCVA was assessed by the nurses monthly using the ETDRS chart at two meters before pupil dilatation. The most recent refractive correction (within 12 months) obtained by subjective refraction was used. BCVA was recorded preoperatively immediately before phacoemulsification; postoperatively at 1 month, 3 months, and 6 months following surgery; and finally at the last visit.

Secondly, in order to identify any change in progression of neovascular AMD, the frequency of anti-VEGF therapy and the CMT before and after cataract surgery was determined. The frequency of anti-VEGF therapy for the two time periods was calculated as the number of intravitreal injections per month. As a control on the effects of maturation, censoring, and mortality, the frequency of anti-VEGF therapy before and after cataract surgery was also compared for the immediate 6 months preceding and following the operation.

CMT was obtained from database immediately before surgery, at 1 month and 3 months after phacoemulsification. We used TopCon 3D OCT-1000 (Tokyo, Japan) Optical Coherence Tomography system to measure CMT. CMT was determined automatically and was analysed by OCT software. The 3D scan protocol was used in this study. Any perioperative complications related to cataract surgery were obtained from database.

All data was collected and entered into spreadsheet software (Excel 2007, Microsoft Corp.). Statistical analysis was performed using the same spreadsheet software and MATLAB r2010a (The MathWorks, Inc.). Comparison of BCVA at the 5 measured time points was performed using the Friedman analysis of variance (ANOVA). Wilcoxon signed rank test was then used to compare each pair of data for BCVA at these 5 measured time points. The Bonferroni correction was applied to any post hoc tests. Comparison of the frequency of anti-VEGF therapy and CMT between the preoperative and postoperative periods was also performed using Wilcoxon signed rank test. A *P* value of < 0.05 was considered statistically significant.

## 3. Results

A total of 30 eyes of 29 subjects were included in this study. 34.5% (10/29) of subjects were male and 65.5% (19/29) of subjects were female. The median age, on the date of cataract surgery, was 84 years (range: from 71 to 93). The median preoperative follow-up period between the first anti-VEGF injection and phacoemulsification surgery was 23.8 months (range: from 4.7 to 46.2), while the median postoperative follow-up period between phacoemulsification surgery and the last visit was 17.5 months (range: from 5.5 to 54.4).

### 3.1. BCVA (LogMAR)

**Figure 1:**
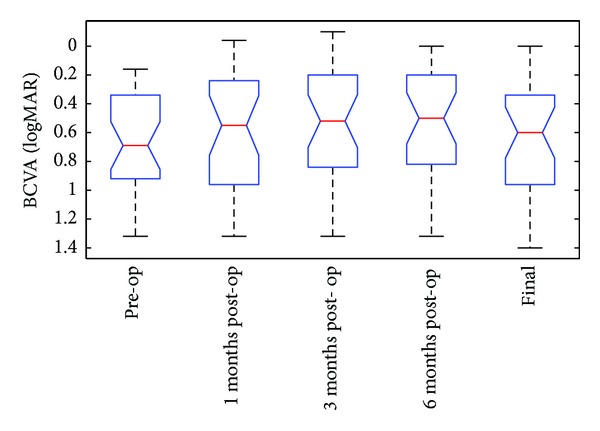
Box-and-whisker plot for best corrected visual acuity (logMAR) at 5 time points.

Table 1 displays the summary statistics for the BCVA readings at 5 measured time points, while Figure 1 shows the box-and-whisker plots for BCVA in logMAR at the same time points. The Friedman analysis of variance (ANOVA) test for the BCVA showed evidence of a significant difference between the 5 time points (*P* = 0.0011). Ad hoc tests (Table 2) indicate that the 3-month and 6-month logMAR readings account for a significant improvement in BCVA from the same readings before surgery.

### 3.2. Frequency of Anti-VEGF Injections

**Figure 2:**
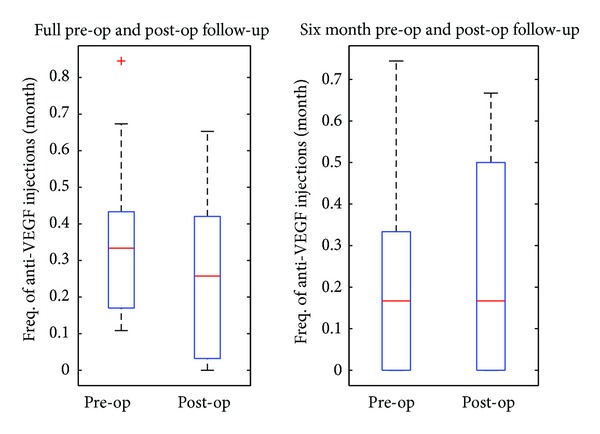
Box-and-whisker plots for the mean monthly frequency of antivascular endothelial growth factor (VEGF) injections for different time periods before and after surgery.

Overall, the median frequency of anti-VEGF injections per month was 0.3335 preoperatively (range: from 0.1082 to 0.8449) and 0.2571 postoperatively (range: from 0 to 0.6527). There is evidence that the frequency of injections was lower after surgery (*P* = 0.0286). As the preoperative follow-up tended to be longer than the postoperative follow-up and the frequency of injections was higher during the initial induction period, the frequency of injections for 6 months pre- and postoperative follow-up periods was compared. The median for both pre- and postsoperative 6-month periods was equal at 0.1667 injections per month (preoperative range: from 0 to 0.7441; postoperative range: from 0 to 0.6667). There was no evidence of a difference in the frequency of anti-VEGF injections between these 2 time periods (*P* = 0.6377).  Figure 2 shows the box-and-whisker plots for the mean monthly frequency of anti-VEGF injections for different time periods before and after surgery.

### 3.3. CMT

**Figure 3:**
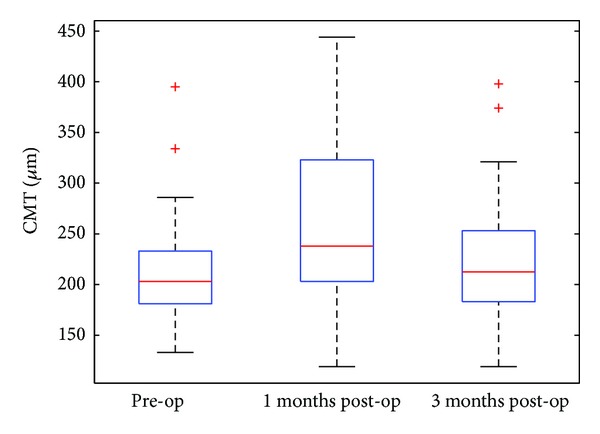
Box-and-whisker plots for central macular thickness (*μ*m) before and after operation.

The median CMT before surgery was 203 *μ*m (range: from 133 to 395), while the same measures 1 month and 3 months following the surgery (±2 weeks) were 238 *μ*m (range: from 119 to 444) and 212.5 *μ*m (range: from 119 to 398), respectively. This data is displayed in Figure 3.

The CMT was significantly higher 1 month following surgery when compared to presurgery values (*P* = 0.0093). On the other hand, there was no evidence of a difference in CMT between before surgery and 3 months after surgery (*P* = 0.3811). Of all the 30 eyes which were in a drug-free phase before surgery, recurrence of leakage requiring anti-VEGF retreatment occurred in 8 eyes (27%) at 1 month, 13 eyes (43%) at 3 months, and 17 eyes (57%) at 6 months after surgery, respectively.

None of the patients included in this study had any perioperative complications recorded during phacoemulsification surgery.

## 4. Discussion

Our results show a beneficial effect of phacoemulsification surgery in eyes with preexisting neovascular AMD treated with intravitreal anti-VEGF injections. There is evidence of an improvement in visual acuity with no increased frequency of anti-VEGF injections to keep the macula fluid free postoperatively. In the current study, there was an overall improvement in the median BCVA after cataract surgery at all postoperative time points (*P* = 0.0011). The most significant improvement in BCVA measured in logMAR units over preoperative values (median = 0.69) was recorded at 3 months (median = 0.52; *P* = 0.0006) and 6 months (median = 0.5; *P* = 0.0005) after surgery (Table 2). When compared to preoperative figures, the BCVA readings after 3 months accounted for a median improvement of 0.12 logMAR units, equivalent to 6 ETDRS letters (range: from −12 to 26), while the BCVA readings after 6 months accounted for a median improvement of 0.14 logMAR units, equivalent to 7 ETDRS letters (range: from −20 to 29). A follow-up period of 3 and 6 months was chosen because this includes the usual 3 months postoperative period following routine cataract surgery when maximal improvement in visual acuity is observed in uncomplicated surgical cases, as can be confirmed by our results (Figure 1).

At final follow-up (median: 17.5 months) there was a decrease in BCVA (median = 0.6) when compared to 6 months after surgery (*P* = 0.0106), even though a gain in visual acuity over preoperative values persisted. However, this was not associated with an increase in the frequency of anti-VEGF therapy postoperatively for both assessed time points (Figure 2). This deterioration in BCVA at the final visit was attributed to macular scar formation rather than exacerbation of exudative AMD. Overall, there was evidence of a significant decrease in the frequency of anti-VEGF injections after surgery. Patients received a median frequency of 0.3335 anti-VEGF injections per month preoperatively compared with 0.2571 per month postoperatively (*P* = 0.0286). In the PrONTO study [22], patients received a median of 5 injections in the first year equivalent to 0.4167 injections per month. The reduced number of injections required after cataract surgery most probably indicates that most subjects were in the remission phase at the time of surgery. These results suggest that cataract surgery does not exacerbate leakage in patients with existing neovascular AMD. No significant difference in the frequency of injections was also obtained when comparing equal pre- and postoperative follow-up periods of 6 months.

A similar improvement in visual acuity following cataract surgery without progression of neovascular AMD was also found in recent studies. In a retrospective case series of 16 subjects with wet AMD, who underwent phacoemulsification, Muzyka-Woźniak [20] obtained a mean BCVA improvement of 3 logMAR lines with no significant difference in the median time interval between injections before and after surgery. At 3 months after surgery, the BCVA of ranibizumab treated eyes in the ANCHOR and MARINA phase 3 trials undergoing cataract surgery improved by a mean of 10.4 (±3.4) letters equivalent to 2 or more logMAR lines when compared to baseline [21]. Similarly, Tabandeh et al. [19] achieved a significant increase in BCVA of approximately 2 logMAR lines from preoperatively to postoperatively in 30 eyes with neovascular AMD who had cataract surgery. Moreover, there was no associated increased frequency of anti-VEGF therapy suggestive of recurrence of leakage from the choroidal neovascular complex postoperatively.

We also used OCT imaging and CMT to assess disease progression after surgery. Retreatment with ranibizumab was performed in the presence of any macular fluid or an increase in CMT of at least 100 *μ*m as detected by OCT, similar to that described in the PrONTO study [22]. Our results show a temporary increase in the CMT at 1 month after surgery over preoperatively (*P* = 0.0093), which returned to baseline at 3 months after surgery (*P* = 0.3811) (Figure 3). However, this was not associated with an increased need for anti-VEGF injections to keep the macula dry postoperatively (Figure 2). It is well known that subclinical cystoid macular edema (CME) occurs in a fair proportion of eyes after uneventful phacoemulsification, even in eyes without any co-existing pathology [23]. OCT evidence of CME following uncomplicated small incision phacoemulsification has been reported between 4% and 24.1% [23–25] but may be as high as 41% [26, 27]. Therefore, we could reasonably postulate that some or most of the eyes had subclinical CME at 1 month, which is supported by spontaneous resolution at 3 months after surgery, which would not be expected in recurrent activation of the choroidal neovascular complex. However, the difference between postoperative CME and reactivation of neovascular AMD may be difficult to determine on OCT without fundus fluorescein angiography, which was one of the limitations of our study. All study eyes were in an injection free phase at the time of cataract surgery. Recurrence of leakage requiring anti-VEGF retreatment occurred in 27% (8/30) of eyes at 1 month, 43% (13/30) of eyes at 3 months, and 57% (17/30) eyes at 6 months after surgery. This appears to be similar to the recurrence rate reported in the PrONTO study.

In our study, there were no complications related to phacoemulsification surgery secondary to multiple previous intravitreal anti-VEGF injections, such as zonular dehiscence, rupture of the posterior capsule, rhegmatogenous retinal detachment, impaired wound healing, wound dehiscence and leakage, corneal edema, or endophthalmitis. Our data indicates a good safety profile and correlates with the findings of Tabandeh et al. [19], Muzyka-Woźniak [20], Jonas et al. [28], and Furino et al. [29].

The limitations of our study include its retrospective design, small sample, short follow-up, and the absence of postoperative fundus fluorescein angiography. Although this is a retrospective review, data was collected prospectively as part of our standard treatment protocol for wet AMD patients. This reduces the weakness of our study, because most retrospective series have a weakness due to variability in data collection and treatment protocols. We did not include a control group which was abandoned for ethical reasons. Hooper et al. [14] showed a 2.1-fold average gain in the quality of life 6 months following cataract surgery in patients with high risk age-related macular degeneration. For us it seemed unreasonable to deprive elderly patients with visually significant cataract of the opportunity of cataract surgery and visual improvement.

In conclusion, our results suggest that phacoemulsification surgery significantly improves the vision in eyes with neovascular AMD and does not appear to worsen disease progression. In addition it appears to be safe, facilitates monitoring of disease progression, and improves the quality of life of this population. Therefore, cataract surgery should be considered in patients with neovascular AMD and visually significant cataract in order to obtain optimal vision in combination with anti-VEGF therapy. Future larger prospective studies with a longer follow-up and use of fluorescein angiography in conjunction with OCT imaging as a predictor of disease progression after surgery should be performed.

## Figures and Tables

**Table 1:** Summary statistics for best corrected visual acuity (logMAR) at 5 time points.

	Pre-op	1 month post-op	3 months post-op	6 months post-op	Final
Median	0.69	0.55	0.52	0.50	0.60
Maximum	0.16	−0.04	−0.10	0.00	0.00
Minimum	1.32	1.32	1.32	1.32	1.40
Mean	0.68	0.59	0.53	0.53	0.62
Standard deviation	0.34	0.37	0.35	0.37	0.39

**Table 2:** *P* values (two-tailed) for comparison of best corrected visual acuity (logMAR) at 5 time points.

	1 month post-op	3 months post-op	6 months post-op	Final
Pre-op	0.0403	0.0006*	0.0005*	0.0872
1 month post-op		0.0180	0.0901	0.3742
3 months post-op			0.8926	0.0100
6 months post-op				0.0106

*Significant.
